# Pamidronate Alters the Growth Plate in the *Oim* Mouse Model for Osteogenesis Imperfecta

**Published:** 2009-12

**Authors:** K. D. Evans, L. E. Sheppard, S. H. Rao, R. B. Martin, A. M. Oberbauer

**Affiliations:** 1 *Department of Animal Science, University of California, Davis. One Shields Avenue, Davis, USA;*; 2 *Orthopaedic Research Laboratories, Research Building I, UC Davis Medical Center 4635 Second Ave., Sacramento, USA*

**Keywords:** pamidronate, *oim*, growth plate, osteogenesis imperfecta, bisphosphonate

## Abstract

Bisphosphonates alleviate bone pain and fractures associated with osteogenesis imperfecta (OI). Using the *oim* mouse model to simulate variations in OI severity, the effect of pamidronate on bone growth was assessed. Homozygous (*oim/oim*) and heterozygous (*oim*/wt) mice from 4 to 12 weeks of age were given pamidronate at 0 mg/kg/wk (control), 1.25 mg/kg/wk (low) and 2.5 mg/kg/wk (high). Humerus and ulna lengths were reduced in *oim/oim* mice relative to those of the *oim*/wt. Further, the *oim/oim* genotype exhibited a 23.5% prevalence of fractures in these bones as compared to the 2.8% prevalence observed in the *oim/*wt mice. Pamidronate tended to reduce fracture prevalence in a dose dependent manner for the *oim/oim* genotype (p<0.08) but had no effect on the low fracture prevalence in *oim/*wtmice. The high dose of pamidronate reduced bone length in females of both genotypes but not males when compared to control (p<0.01). Pamidronate increased growth plate area (p<0.05) by increasing growth plate height, particularly the proliferative and hypertrophic zones, in both genotypes indicating reduced growth plate cell turnover. The increased area coincided with increased osteoclast numbers in the metaphyseal region (p<0.05) though when corrected for the greater mineralized surface area that accompanies bisphosphonate treatment, osteoclasts per surface area were reduced indicating reduced resorptive capacity. This study demonstrated that the effects of pamidronate were independent of the degree of collagen deficit and fracture prevalence was improved in the most severe OI model, the *oim/oim* genotype.

## INTRODUCTION

Osteogenesis imperfecta (OI) is a genetic disorder of bone fragility caused by defects in either the pro-α1(I) (COL1A1) or pro-α2(I) (COL1A2) genes encoding type I collagen. Phenotypic expression is highly variable with OI patients suffering varying degrees of impairment in bone ultrastructure and mineralization, increased microfractures and tissue fragility, accelerated remodeling, osteoporosis and atraumatic fractures (1). The variable phenotypic expression and mode of inheritance led to the classification of ten clinical subtypes (2) of which OI Type III is the most severe with structural alterations of the collagen molecule. Less severe OI phenotypes reflect mutations that alter the quantity of collagen secreted (3). The *oim/oim* mouse represents a model for OI as it has a single nucleotide deletion in the *Cola-2* gene preventing the formation of normal type I procollagen (4).

Bisphosphonates decrease bone remodeling and fracture rate in post-menopausal osteoporosis (5) and they are used in the treatment of OI. Substantial improvements have been observed in juvenile OI patients treated with pamidronate (an aminobisphosphonate). In controlled clinical studies, cyclic pamidronate infusions decreased bone pain, increased height, and increased bone mineral density (reviewed in 6).

In the OI condition overall bone length and stature are reduced. There are two mechanisms postulated for the observed decreased bone length: frequent fractures and inherent abnormalities in the turnover of cartilage to bone at the growth plate (7). Bisphosphonates affect bone remodeling through inhibition of the osteoclast and thereby reduce fracture incidence. Bisphosphonates also inhibit osteoclast action including that responsible for bone turnover at the chondro-osseous junction. Studies of normal mice have suggested reduced growth plate turnover and osteoclast activity at the growth plate in response to bisphosphonate treatment (8), thus concerns exist for children receiving bisphosphonates regarding the consequences of suppressing normal bone modeling (6).

Given the differing degrees of severity seen in the OI condition, the magnitude of collagen defect may respond differentially to bisphosphonate treatment. The present study evaluated whether bisphosphonate treatment would affect growth parameters differently depending upon the extent of the collagen I deficiency. This study was undertaken to determine if prolonged cyclic dosing of pamidronate, a common clinical bisphosphonate, had differential effects on linear bone growth and growth plate parameters in a mouse model that simulates different degrees of collagen deficits.

## MATERIALS AND METHODS

### Animal breeding and genotyping

Heterozygous B6C3Fe-*a/a-Cola2^oim/+^* hybrid *oim*/wt breeder animals were obtained from Jackson Laboratories (Bar Harbor, ME) and housed according to current NIH guidelines. All mice were kept at constant temperature (70 ± 2°F) and fed Purina mouse diet formula 5008 (Purina, St. Louis, MO) and tap water ad lib. All animal protocols were approved by the University of California Davis Institutional Animal Care and Use Committee. Animals were weaned at 3 weeks of age and housed according to sex. All animals were genotyped by polymerase chain reaction as previously described (9) with groups of 9–10 animals assigned randomly to 12 experimental groups based on sex (male and female), genotype (*oim/oim, oim*/wt), and pamidronate dose. Pamidronate (Aredia™, Novartis Pharmaceuticals, East Hanover, NJ) was reconstituted in phosphate-buffered saline (PBS) and administered weekly by intraperitoneal injections beginning at 4 weeks of age (±2 days), and for 8 weeks thereafter. Animals were given PBS vehicle alone (vehicle control (0 mg/kg/wk) or pamidronate at low (1.25 mg/kg/wk) or high (2.5 mg/kg/wk) dose; these doses were selected to bracket the dose given to children with bone abnormalities including OI (10–12). At 12 weeks of age, mice were killed by CO_2_ narcosis and their humerus and ulna bones collected.

### Bone length measurements

The left and right humerus and ulna were dissected from each animal and placed in 10% formalin for a period of at least 1 week. The bones of each limb were cleaned of adherent tissues and their lengths measured using calipers accurate to 0.1 mm (Manostat Inc., Rennes, Switzerland). The humerus was measured from the proximal end of the humeral head to the distal humeral epicondyle. The ulna was measured from the proximal end of the olecranon process to the distal ulna junction with the carpus as previously described (13). Fractures, as indicated by fracture callous or observed fractured bone upon dissection, were recorded for the humeri and ulnae from each animal.

### Tissue preparation for histology

The left humerus from 5 animals in each group was drained free of formalin and decalcified in 0.5 M EDTA for at least 3 weeks. After decalcification, each bone was individually rinsed for 72 h under running water and stored in 70% ethanol for a minimum of 24 h. Tissue dehydration and processing were done with a VIP tissue processor (Tissue-Tek VIP 1000/2000, Miles Scientific, Naperville, IL). The bones were then embedded in paraffin for the cutting of longitudinal sections (LKB Bromma rotary microtome) suitable for determining the area, diameter, height, and gross histological appearance of the proximal growth plates as well as TRAP (tartrate resistant acid phosphatase) staining of osteoclasts. Sections were dried in a 37°C oven for at least 2 days prior to tissue staining.

### Growth plate area, diameter, height measurements

Longitudinal sections (6 μm) were stained with hematoxylin and eosin, viewed under a compound light microscope (Nikon SK, Tokyo Japan) at a magnification of 400×, and photographed using a Nikon Coolpix 3300 camera linked to a computer with Nikon View version 6.2.1 for image conversion (Nikon, Garden City, NY). Images were analyzed for growth plate area, diameter, and height using Sigmascan 4.0 (SPSS Scientific, Chicago, IL). Growth plate measurements and gross comparisons were taken as previously described (13). Cell numbers within each humeral growth plate zone were also counted in three consecutive sections for *oim/oim* mice treated with pamidronate or vehicle control.

### TRAP Staining of osteoclast cells

Longitudinal sections (6 μm) of the left humerus bone for each sex, genotype, and dose combination were rehydrated followed by immersing the bone sections in a reactivation buffer (0.07 M Tris solution and 0.06 M hydrochloric acid) overnight at a pH of 9.0 (14). TRAP staining was done according to previous studies (14) with the slides then rinsed with deionized water and counterstained with 1% methyl green (Sigma-Aldrich Inc., St. Louis, MO, USA). TRAP positive osteoclasts on three consecutive bone sections from the left humerus of each animal were counted and averaged to quantify the number of osteoclasts for each of four regions of the growth plate. These regions included the epiphysis proximal to the superior growth plate, the metaphysis immediately distal to the superior growth plate, the ventral metaphysis including the area from the last intact transverse cartilage septa distally to the end of the trabeculae, and the diaphysis from the trabeculae distal to the contralateral ventral metaphysis. Thus, for each animal, each region’s osteoclast number was an average of three recordings. Total osteoclast and TRAP positive cell numbers for each region were taken using an ocular eyepiece grid at a magnification of 400× (0.0004 mm^2^) that encompassed the entire growth plate plus 0.02 mm both distally and medial-laterally. Calcified cartilage and mineralized bone surface areas for the metaphysis were also determined to correlate differences in osteoclast number per surface area to correct for surface area changes as a result of pamidronate treatment.

### Statistical analysis

Bone length data were analyzed for male and female mice striated by genotype and pamidronate dose for both humerus and ulna by least squares analysis of variance (PROC GLM, Procedure General Linear Model, SAS version 6.07; SAS Institute Inc., Cary, NC) with fixed effects of genotype, dose, and genotype by dose interaction. Within an individual, bone lengths were not significantly different between the right and left sides (p>0.2 for humerus and for ulna), therefore the right and left bone lengths were averaged for each animal. The growth plate area, diameter, height, and the number of TRAP stained osteoclast cells per treatment were analyzed by least squares analysis of variance, using PROC GLM with fixed effects of genotype, dose, and genotype by dose interaction. Post-hoc comparisons used a Bonferroni adjustment for multiple comparisons. For all analyses, significance was defined as p<0.05, with p<0.10 defined as tending toward significance.

## RESULTS

### Bone Length

The *oim/oim* mice had reduced humerus and ulna bone lengths in both sexes relative to *oim*/wt mice (p<0.001) with *oim/oim* mice exhibiting a 4% reduction in length (Table 1). While pamidronate dose did not alter bone length in males, pamidronate reduced bone length of the humerus and ulna in female mice regardless of genotype (p<0.05) with no significant genotype by dose interaction in either sex (p>0.2).

**Table 1:** Pamidronate dose effects on humerus and ulna bone lengths (mm) in *oim/oim* and *oim*/wt male and female mice

MALES	Genotype (p<0.0001)	
**Humerus**	***oim/oim***	***oim*/wt**
Vehicle Control	11.7 ± 0.1	12.5 ± 0.1
Low Dose	11.8 ± 0.1	12.2 ± 0.1
High Dose	11.8 ± 0.1	12.2 ± 0.1
**Ulna**		
Vehicle Control	13.7 ± 0.1	14.2 ± 0.1
Low Dose	13.5 ± 0.1	14.4 ± 0.1
High Dose	13.8 ± 0.1	14.2 ± 0.1
**FEMALES**	**Genotype (p<0.0001)**	

**Humerus**	***oim/oim***	***oim*/wt**
Vehicle Control	11.9 ± 0.1^a^	12.3 ± 0.1^a^
Low Dose	11.7 ± 0.1^a^^b^	12.0 ± 0.1^a^^b^
High Dose	11.5 ± 0.1^b^	11.7 ± 0.1^b^
**Ulna**		
Vehicle Control	13.6 ± 0.1	14.2 ± 0.1^a^
Low Dose	13.4 ± 0.1^c^	14.0 ± 0.1^a^^b^
High Dose	13.4 ± 0.1^c^	13.7 ± 0.1^b^

Values are expressed as means ± standard error of the means. Pamidronate doses were control (0 mg/kg/wk), low (1.25 mg/kg/wk), and high (2.5 mg/kg/wk) given for 8 weeks; n=18 bones for each group.

a, b Superscripts designate significantly different means within a genotype, p<0.05;

c denote difference at p<0.1.

The decrease in female bone length with pamidronate could have been a result of reduced bone fracture and altered bone modeling in response to the anti-resorptive properties of pamidronate. The *oim/oim* genotype as a whole exhibited a combined 23.5% prevalence of fractures in the humeri and ulnas as compared to the 2.8% prevalence observed in the *oim/*wt mice (p<0.001). Pamidronate tended to reduce fracture prevalence in a dose dependent manner for the *oim/oim* genotype regardless of sex (30 ± 4%, 26.3 ± 4%, and 14.5 ± 4%, for vehicle control, low, and high pamidronate doses, respectively; p<0.08) but did not alter the fracture prevalence for the *oim/*wtgenotype (2.8%). Within the ulna, the majority of fractures involved the olecranon process of the ulna, whereas in the humerus the majority of fractures occurred in the mid-diaphyseal region,

### Growth plate area, diameter, height measurements

Most long bone growth occurs by cell turnover and matrix deposition at the endochondral growth plates. Therefore, the effects of genotype and pamidronate dose were evaluated for their effect on the volume of the growth plate (Table 2). Growth plate areas of the *oim/oim* mice were greater than the *oim/*wt mice by ∼30% (p<0.01) in vehicle control treated animals. Pamidronate increased growth plate area by an average of ∼16 % in the low dose and 29% in the high dose compared to that observed for the vehicle control mice. The greatest increase in area was observed in the high pamidronate dose given to male *oim/*wt mice in which the growth plate area increased 67% as compared to the 12% increase in area observed with the same dose given to the *oim/oim* animals (p<0.05). This increase in growth plate area was due solely to an increase in growth plate height and not diameter (Table 2). Further, the increased height corresponded to greater proliferative and hypertrophic zone cell numbers in response to the pamidronate treatment. The high dose increased the number of chondrocytes in the proliferative and hypertrophic zones by 33% and 23%, respectively over vehicle control (p<0.05, data not shown).

**Table 2:** Genotype and pamidronate dose effects on humerus growth plate area, length, and diaphyseal width

	Growth plate area (mm^2^)		Growth plate average height (mm)		Growth plate average diameter (mm)	
**MALES**	** **		** **		** **	
**Pamidronate**	**Genotype (p<0.01)**		**Genotype (p<0.4)**		**Genotype (p<0.3)**	
	***oim/oim***	***oim*/wt**	***oim/oim***	***oim*/wt**	***oim/oim***	***oim*/wt**

Vehicle Control	0.378 ± 0.25	0.276 ± 0.25^a^	0.14 ± 0.02^a^	0.11 ± 0.02^a^	2.21 ± 0.06	1.96 ± 0.06
Low Dose	0.457 ± 0.25	0.361 ± 0.25^a^^b^	0.23 ± 0.02^b^	0.22 ± 0.02^b^	2.10 ± 0.06	1.99 ± 0.06
High Dose	0.424 ± 0.25	0.460 ± 0.25^b^	0.24 ± 0.02^b^	0.24 ± 0.02^b^	1.98 ± 0.06	2.06 ± 0.06
**FEMALES**	** **		** **		** **	
**Pamidronate**	**Genotype (p<0.04)**		**Genotype (p<0.6)**		**Genotype (p<0.5)**	
	***oim/oim***	***oim*/wt**	***oim/oim***	***oim*/wt**	***oim/oim***	***oim*/wt**

Vehicle Control	0.385 ± 0.27	0.300 ± 0.27	0.13 ± 0.02^a^	0.13 ± 0.02^a^	2.09 ± 0.07	1.91 ± 0.07
Low Dose	0.351 ± 0.27	0.366 ± 0.27	0.20 ± 0.02^b^	0.21 ± 0.02^b^	1.88 ± 0.07	2.06 ± 0.07
High Dose	0.439 ± 0.27	0.365 ± 0.27	0.23 ± 0.02^b^	0.21 ± 0.02^b^	2.08 ± 0.07	2.03 ± 0.07

Three doses of pamidronate (control (0 mg/kg/wk), low (1.25 mg/kg/wk), and high (2.5 mg/kg/wk)) were given for 8 weeks to *oim/oim,* and *oim*/wt male and female mice. Values are expressed as means ± SEM (n = 5 mice per group);

a, b means with different superscripts within genotype differ significantly.

### Osteoclast cell numbers

The total number of osteoclasts exhibiting TRAP staining was increased in the *oim/oim* mice relative to the *oim*/wt mice in both sexes: 858.8 ± 63.1 vs. 662.0 ± 57.3 (p<0.03) for males and 816.1 ± 63.0 vs. 656.1 ± 68.8 (p<0.1) for females for *oim*/*oim* and *oim*/wt, respectively. The increased total number of TRAP stained osteoclasts in all cases was due to increased osteoclasts in the metaphysis immediately distal to the superior growth plate and the ventral metaphysic; these regions accounted for approximately 65% of the TRAP stained osteoclasts (Table 3). The number of TRAP stained osteoclasts in the regions of the epiphysis (203.2 ± 29.9 and 225.9 ± 29.9, males and females, respectively) and diaphysis (10.6 ± 2.8 and 13.9 ± 4.1, males and females, respectively) were unchanged with genotype or pamidronate dose. Though metaphyseal osteoclast numbers in both sexes and both genotypes displayed increases with increasing pamidronate dose (pamidronate dose effect, p<0.05) only the *oim*/wt increase in males was significant when considering a genotype by dose interaction (p<0.05).

**Table 3:** TRAP stained osteoclast cell numbers (metaphyseal and ventral metaphyseal) at the humerus growth plate

	Metaphyseal osteoclast numbers		Metaphyseal Osteoclasts corrected for bone surface area	
**MALES**	** **		** **	
**Pamidronate**	**Genotype (p<0.03)**		**Genotype (p<0.7)**	
	***oim/oim***	***oim*/wt**	***oim/oim***	***oim*/wt**

Vehicle Control	527.0 ± 55.4	258.3 ± 71.6^a^	21.4 ± 2.5b	15.0 ± 2.5
Low Dose	484.6 ± 55.4	546.0 ± 62.0^b^	7.5 ± 3.0^a^	10.3 ± 2.5
High Dose	781.5 ± 87.6	662.2 ± 55.4^b^	9.4 ± 1.8^a^	13.6 ± 1.7
**FEMALES**				
**Pamidronate**	**Genotype (p<0.1)**		**Genotype (p<0.2)**	
	***oim/oim***	***oim*/wt**	***oim/oim***	***oim*/wt**

Vehicle Control	449.6 ± 69.9	245.2 ± 69.9	14.2 ± 1.8	17.2 ± 1.8
Low Dose	597.3 ± 78.1	475.6 ± 69.9	9.2 ± 1.8	12.0 ± 1.6
High Dose	586.3 ± 78.1	508.3 ± 90.2	12.0 ± 1.2	11.2 ± 1.4

Genotype and pamidronate dose effects on total TRAP stained osteoclast cell counts within the metaphysis and ventral metaphysis. The total metaphyseal TRAP stained cells were corrected for calcified cartilage and mineralized bone surface area within the metaphyseal region to account for differences in surface area as a result of the pamidronate treatment. Three doses of pamidronate (control (0 mg/kg/wk), low (1.25 mg/kg/wk), and high (2.5 mg/kg/wk)) were given for 8 weeks to *oim/oim* and *oim*/wt male and female mice. Values are expressed as means ±SEM (n = 5 animals per group);

a, b means with different superscripts within genotype differ significantly.

When the total number of metaphyseal TRAP stained osteoclasts was corrected for metaphyseal bone surface area, pamidronate dose significantly decreased the number of osteoclasts on a per surface area in both males and females (p<0.05) with only a significant genotype by dose effect seen in male *oim/oim* mice. The data revealed that the greater total number of osteoclasts detected with increasing pamidronate dose was actually reduced when evaluated as a proportion of osteoclasts on the surface area suggesting that the overall mineralized surface area was greater in response to pamidronate treatment and the number of active osteoclasts were diminished.

## DISCUSSION

In this mouse model of varying severity of OI, the *oim/oim* mice had shorter bones than the less severe *oim*/wt genotype consistent with previous findings (13). The pamidronate administered in this study resulted in shorter bones (females) having a larger growth plate area and height with increased proliferative and hypertrophic cell numbers. These data support the hypothesis that long term cyclic pamidronate treatment reduces cell turnover at the endochondral growth plate.

Pamidronate is used to decrease fracture incidence and bone pain and thus improve the quality of life for OI patients (15). In the present study, pamidronate tended to reduce fracture incidence in a dose dependent fashion within the *oim/oim* mice. As reported in previous studies, bones of *oim*/wt mice have structural and material properties intermediate to normal wild type and *oim/oim* mice (4, 16). The *oim*/wt heterozygous animals of the present study had few fractures with the vehicle control treatment consistent with those reports. Exposure to pamidronate did not reduce fracture prevalence in the *oim*/wt mice that had only moderately compromised bone properties.

It is known that bisphosphonates gain access to the growth plate (17, 18). The inhibitory effect of bisphosphonate within the growth plate on osteoclast function (10, 19) would likely impede invading vasculature into the area of the chondro-osseous junction thereby reducing hypertrophic chondrocyte turnover. Bisphosphonates have been shown to inhibit VEGF (vascular endothelial growth factor) signaling (20) which would also lead to a decrease in vascular penetration into the ventral metaphysis. The inhibition of the vascular invasion at the terminal hypertrophic zone would result in an increased growth plate height and reduced cell turnover and diminished modeling of the cartilage to trabecular bone at the metaphyseal junction. When these effects combine, the growth plate would increase in height but have diminished contribution to linear bone growth as seen in the present study.

Whereas the vast majority of human or animal bisphosphonate studies do not consider gender effects, treatment with pamidronate in the present study demonstrated differential gender response. Bone length was not altered in males treated with pamidronate but diminished overall bone lengths were seen for females with the greatest reductions detected for the high dose of pamidronate and in the *oim*/wt genotype. Male mice mature at earlier ages than females (21) with the ulna and humerus reaching mature size by 49 days of age (22). Initiation of pamidronate in the current study was past the maximal bone growth rate in males and therefore the effects on bone growth would be diminished relative to females.

In our previous work investigating alendronate (13), a bisphosphonate having much greater potency than pamidronate (23), male mice exhibited reduced bone length. The higher resorptive potency of alendronate than pamidronate may account for the different observations for males between the previous and the present study. The low potency of the pamidronate may have been inadequate to exert a detectable effect on bone that was in the deceleration growth phase. In addition, bisphosphonates are preferentially sequestered in mineralized tissue (23); males have a greater amount of metaphyseal trabecular bone relative to females (24) thereby permitting a reservoir of alendronate adjacent to the growth plate. In contrast, female *oim/oim* mice exposed to alendronate did not show a reduction in bone length whereas lengths were reduced in the present pamidronate study. Alendronate has a higher clearance rate than pamidronate (23) and limited trabecular bone below the growth plate for alendronate sequestration could have combined to reduce the effect of alendronate in the female.

The comparable height increases for growth plates of both sexes treated with pamidronate suggest bone turnover was altered equally. However, because the growth plates were at different functional maturities the effect on linear bone growth would differ by sex. Growth plates persist beyond their functional contribution to bone elongation (25) and for earlier maturing males the moderate reduction of turnover by pamidronate would minimally impact elongation. In females the reduced turnover would be expected to inhibit bone elongation. The gender differences seen in the present study also may reflect an interaction of the pamidronate with the hormonal milieu of females. The gender response to bisphosphonate treatment and genotypic interaction needs further exploration.

The increased number of TRAP positive multinucleated osteoclasts in the metaphysis and ventral metaphysis of pamidronate treated animals in the present study may indicate varied actions of pamidronate on osteoclast function. The greater overall number of osteoclasts yet reduced numbers when expressed as per mineralized surface area confirmed that osteoclast function was limited by pamidronate treatment. Further, observational evaluation of the TRAP positive multinucleated cells in the present study revealed that among the pamidronate treated animals there were relatively fewer nuclei per osteoclast than controls (data not shown). Thus, though the absolute numbers increased, the osteoclasts observed were less mature and had reduced resorptive function.

Overall osteoclast numbers were similar between the two sexes but when corrected for surface area, the numbers differed for pamidronate treated *oim/oim* males and females. Untreated *oim/oim* males should have a high degree of osteoclastic resorption, especially given the greater metaphyseal trabecular volume found in males relative to females (24), as the modeling process attempts to replace the defective collagen matrix,. Therefore, inhibiting the calcified cartilage and mineralized bone resorption would accentuate the differences observed for the two sexes as the male trabecular volume would be greater overall.

The fewer osteoclast numbers in the *oim*/wt animals relative to the *oim/oim* animals corresponds to the reduced structural strength of the *oim/oim* mice relative to the *oim*/wt animals which represent a less severe OI model (4, 16). The less severe impact on bone properties of the *oim*/wt genotype would require less modeling/remodeling activity than the more severe defect induced by the homozygous *oim/oim* genotype. However, following exposure to pamidronate, the effect was similar for both genotypes: an increased number of osteoclasts with apparent reduced resorptive function indicating that pamidronate therapy would be effective in most OI conditions.

Several studies have shown that cyclic pamidronate administration to children with OI for treatment periods of up to 6 years does not alter growth (26, 27) consistent with the findings for males in this study. In contrast, some studies have shown evidence of a temporary growth alteration associated with pamidronate treatment in the form of distinct radiodense sclerotic lines in the metaphyseal region (28, 29). A recent review of clinical bisphosphonate trials urges additional research to clarify some of the discrepant outcomes reported in the literature (6). Decreasing chondrocyte turnover and inhibiting osteoclasts would lead to a buildup of defective mineralized type I collagen laid down in the area of the metaphysis and chondro-osseous junction. This effect could make those bones more brittle over time due to the change in the biochemical makeup of the bone (4, 16, 30). The reduced turnover at the chondro-osseus junction seen in the present study would support the concern of accumulation of defective mineralized collagen as well as suggesting that altered modeling would also occur in a normal animal treated with pamidronate.

In addition to being clinically relevant for OI treatment, bisphosphonates are effective in improving bone integrity and decreasing pain associated with bone metastases (31) or other juvenile cancers (32). In a number of human studies, bisphosphonate treatment leads to abnormal bone remodeling and accumulation of mineralized cartilage remnants in growing individuals (6, 28, 33). Understanding the consequences of bisphosphonate treatment on bone elongation will enable appropriate therapeutic applications in a number of conditions. The present study demonstrated a gender interaction with pamidronate with bones from females more sensitive to the endochondral growth inhibiting effects of the drug suggesting additional investigation is warranted. Although pamidronate did not alter the low prevalence of fractures in the *oim*/wt genotype, the data demonstrated that the effects of pamidronate were similar across genotypes indicating a generalized effect on bone parameters regardless of the severity of the OI phenotype.
